# Supplementary material for: Diagnostic Performance of Publicly Available Large Language Models in Corneal Diseases: A Comparison with Human Specialists
Source: Diagnostics (Basel). 2025 May 13;15(10):1221. doi: 10.3390/diagnostics15101221 (PMC12110359; doi:10.3390/diagnostics15101221)
Supplement: Supplementary file 1 [file diagnostics-15-01221-s001.zip › diagnostics-3569508-supplementary.pdf]

Case number 1:

A 39-year-old white male contact lens wearer is referred to the clinic with cloudy vision, photophobia, and a red, painful right eye. The patient is a contact lens wearer who has used disposable soft contact lenses for the past 3 months. Three weeks prior to presentation at the clinic, the patient began to develop cloudy vision, photophobia and increasingly exquisite pain in the right eye (OD). Pain increased to 8 out of 10 on a pain scale, despite topical antiobiotic therapy at an outside facility. When the patient presented to the clinic, he was on Gatifloxacin and Tobramycin drops every hour and cyclopentolate 1% twice a day (BID), OD. Despite this therapy, the ulcer in the right eye persisted and was worsening. External and anterior segment examination are the following: OD: 4x4 mm stromal ring infiltrate with surrounding white blood cell (WBC) infiltration. Keratic precipitates line the endothelium inferior to the ring infiltrate and there is a 0.5mm hypopyon. Small defects in the epithelium are present over the area of ring infiltrate. Enlarged corneal nerves (radial perineuritis) are also seen on high magnification. There is 4+ conjunctival injection and the anterior chamber is filled with 3+ cells and 2+ flare reaction. OS: Normal Dilated fundus exam (DFE): Very difficult and hazy posterior view, OD. Normal disc, macula, vessels, and periphery, OS. what is most likely the diagnosis?

Case Number 2:

A 51-year-old male with keratoconus and history of rigid gas permeable (RGP) contact lens wear in the left eye presented with left eye redness, pain, light sensitivity, and tearing for 1 week. He was initially seen at an acute care clinic where he was told he had scratched his conjunctiva. He then presented to his regular ophthalmologist who found him to have acute corneal edema and referred him to the University of Iowa Hospitals and Clinics (UIHC). He was started on ciprofloxacin and hypertonic saline drops by the outside provider. At the time of his presentation, his pain was rated as 7 out of 10 and described as a constant surface irritation and scratchy sensation. He described constant tearing and blurry vision which did not change throughout the day. The patient took out his contact lens when symptoms began. slit lamp examination showed in left eye Inferior conical protrusion of cornea, focal area of massive inferior corneal edema with overlying microcystic edema and bullae, epithelium intact, no infiltrates or keratic precipitates. what is most likely the diagnosis?

Case Number 3:

A 52-year-old Caucasian male presents to the cornea clinic with a chief complaint of vision loss. In addition to his vision loss, he complains of itchy eyes and mucoid discharge. He also reports a history of waxing and waning eczema over the past decade. Two years prior to this visit, he presented with similar symptoms and was treated with medroxyprogesterone and fluorometholone (FML) three times

a day in both eyes. This medical regimen improved his symptoms. Unfortunately, the patient discontinued his ocular prescription and was lost to follow-up after the initial visit. Past Ocular History: Unremarkable for surgery or trauma. Medical History: History of uncontrolled eczema for the past decade. Medications: He uses artificial tears as needed. Otherwise, he takes no medications or other eye drops. Family History: Father with cataract. No other pertinent family history. Social History: The patient is a habitual smoker (1 pack per day). Ocular Examination: Visual Acuity, with correction: Right eye (OD): 20/60-1 Left eye (OS): 20/70-1 Extraocular motility: Full, both eyes (OU) Pupils: Briskly constrict from 5mm in dark to 3mm in light. No relative afferent pupillary defect. Confrontation Visual Fields: Full OU. Intraocular pressure: OD — 13 mmHg; OS — 12 mmHg Slit lamp examination: Lids: Hypertrophy, hyperpigmentation, and erythema of the lid skin. Cicatricial ectropion of the lower lid. Near entire lower lid madarosis with rolled scarred lid margin. Conjunctiva/sclera: Fornix foreshortening and symblepharon of the conjunctiva with 2+ diffuse injection, more prominently OD (figure 1E). Cornea: Near complete conjunctivalization of the cornea with haze entering the visual axis inferonasally (figures 1C and D). Anterior chamber: Deep and quiet. Iris: Normal Lens: 2+ nuclear sclerosis and 2+ posterior subcapsular cataract OU (figure 1F). Dilated Fundus Exam: Hazy view to posterior pole due to conjunctivalization and posterior subcapsular cataract but appears normal OU. what is most likely the diagnosis?

Case Number 4:

A 71-year-old female was referred for evaluation of a corneal scar in the right eye (OD). Ten years previously she had been informed that the scar was present when she was evaluated for painless and slowly progressive vision loss. She attributed the scar to a "fan injury" that she experienced at 20 years of age. Over the last ten years, she has experienced further painless vision loss OD. The vision in the left eye (OS) has been excellent. Past Ocular History: "Corneal scar," OD Non-exudative age-related macular degeneration, both eyes (OU) Cataracts, OD worse than OS No history of ocular surgery Past Medical History: Hypertension No known history of endocrine abnormalities, renal dysfunction, or autoimmune disease Medications: Lisinopril, AREDS 2 vitamin Allergies: No known drug allergies Family Ocular History: Non-contributory Social History: Former smoker with 25 pack-year history Review of Systems: All systems negative Visual acuity with correction, distance: OD: Counts fingers at 1 foot OS: 20/20 -2 Intraocular pressure (Applanation): OD: 16 mmHg OS: 16 mmHg Current spectacle prescription: OD: -0.25 +0.50 x 147, Add: +2.50 OS: +3.25 sphere, Add: +2.50 Confrontation Visual Fields: Full Pupils: No relative afferent pupillary defect OU Extraocular Motility: Full OU Pachymetry: OD: 619 microns OS: 582 microns Keratometry (NIDEK): OD: 46.42 D @ 25 degrees, 45.18 D @ 115 degrees OS: 44.06 D @ 174 degrees, 43.10 D @ 84 degrees External exam: Normal Anterior segment exam: Lids: Normal OU Conjunctiva/Sclera: Clear and quiet OU Cornea: Band-shaped, horizontal, gray-white subepithelial corneal opacity in the interpalpebral fissure with involvement of visual axis OD; normal OS Anterior chamber: Deep and quiet OU Iris: Normal OU Lens: Nuclear sclerotic cataract more prominent OD than OS Vitreous: Normal OU Retina: Inadequate view OD; non-exudative age-related macular degeneration OS. what is the most likely diagnosis?

Case Number 5:

A 42-year-old Caucasian female complains of an annoying red left eye for the past two months. On occasion, the left eye has been "aching" with a "pressure sensation". Despite a course of antibiotics to treat a suspected sinusitis, her symptoms persisted and gradually worsened with time.

In the past month she also developed vertigo, nausea, otalgia, diminished appetite, as well as progressively worsening vision. Acetaminophen provided moderate relief from her pain. She denied any hearing loss, diplopia, fever, cough, rhinorrhea, otorrhea, tinnitus, and vomiting. She presented to Clinic in the spring of 2007 after having been seen by several doctors and frustrated with her persisting and progressive symptoms. she has History of hypothyroidism, Type II diabetes mellitus, nonalcoholic steatohepatitis, polycystic ovarian sydrome, uterine fibroids, fibromyalgia, Celiac's disease, and borderline hypertension. She also had history of taking these medications such as Trazodone, lorazepam, meperidine, levothyroxine, albuterol, fluoxetine, montelukast, and cyclobenzaprine. Additionally, the patient periodically takes a daily multivitamin, fish oil, acetaminophen, and calcium and magnesium supplements. Ocular Examination showed Moderate photophobia during the exam. here Visual Acuity was 20/20 in Right eye; 20/20 in Left eye Pupils: No relative afferent pupillary defect (RAPD) Intraocular pressure: Normal, OU Slit lamp examination: Limbal edema and stromal opacity were noted as well as several fine vascular loops extending into the mid-stroma of the cornea. There is mild vascular injection, especially inferiorly and at the episclera covered by the lower lid, associated with tenderness of the globe. **what is the most likely the diagnosis?**

Case Number 6:

A 45 year-old male was referred to the UIHC ophthalmology department with concern for possible corneal ulcer of the left eye. His symptoms began three weeks prior with irritation, tearing, and redness. He presented to urgent care shortly after developing symptoms and was diagnosed with conjunctivitis of the left eye; he was treated with polymyxin B/trimethoprim (Polytrim) drops (1 drop in left eye every 4 hours for 5 days). He used the drops as directed and his symptoms resolved. A week later, he had recurrence of symptoms with redness of the left eye followed by irritation, pain, photophobia, and blurry vision. He also developed clear discharge with mattering in the morning. He tried using the leftover Polytrim drops and artificial tears, but with no benefit. He presented to urgent care and was referred to the UIHC ophthalmology clinic for further evaluation. He denied any problems with the right eye, previous episodes of recurrent conjunctivitis, or any symptoms of chronic dry eye in either eye. Other review of systems was only pertinent for recent nasal congestion. Slit lamp examination showed: OD Lids/Lashes: blepharitis with collarettes Conjunctiva/sclera: 1+ conjunctival injection Cornea: Clear Anterior Chamber: Deep and quiet Iris: Normal architecture Lens: Clear Vitreous: Normal OS Lids/Lashes: erythematous and edematous lid Conjunctiva/sclera: 3+ conjunctival injection most prominent nasally, mild chemosis Cornea: circumlimbal opacity extending from 8 to 12 o'clock with overlying epithelial defect (1.5mm wide extending from 8:30- 11:00) that is 0.5-1.0mm from limbus; there is an inferonasal area of infiltrate with no overlying epithelial defect. Corneal edema is present nasally with Descemet's membrane folds. There is vessel encroachment from the limbus from 5:00-1:00. Anterior Chamber: Deep and quiet Iris: Normal architecture Lens: Clear Vitreous: Normal. His IOP was normal and his VA was 20/20 in Right eye (OD), Left eye (OS): 20/40-1, pinhole to 20/20-1. what is the most likely diagnosis?

Case Number 7:

A 4-year-old girl with failure to thrive, severe photophobia, and has always been in the lower 1/3 percentile for growth and development. Parents have noted child becoming increasingly photophobic. Child was recently admitted for "severe illness" and was diagnosed with renal insufficiency. she has No previous ocular or health problems. ocular examination showed: Vision 20/80 OU with line pictures.

IOP 16 mmHg OU EOM Full OU DFE normal OU SLE notable for crystalline stromal deposits in the cornea from limbus-to-limbus OU. what is the most likely diagnosis?

Case Number 8:

A 17-year-old boy presented with one day of acute-onset bilateral foreign body sensation, tearing, photophobia, blurriness, and redness. His symptoms were worse at the end of the day. He denied ocular itching, discharge, contact lens use, known foreign matter exposure, history of upper respiratory illness, sick contacts, and cold sores. One week prior to presentation, he was admitted to the hospital for treatment of acute myeloid leukemia (AML). His chemotherapy regimen included cytarabine, which he had received four days prior to the onset of symptoms. on slit lamp examination showed: right corneal Tiny intraepithelial microcysts scattered diffusely and 1+ punctate epithelial erosions scattered diffusely, and conjunctival 1+ injection without chemosis, follicles, or papillae. left eye showed corneal Tiny intraepithelial microcysts scattered diffusely and 1+ punctate epithelial erosions scattered diffusely, and conjunctival 1+ injection without chemosis, follicles, or papillae. what is the most likely diagnosis?

Case Number 9:

The ophthalmology service at the University of Iowa Hospitals & Clinics was consulted for a 16-year-old male inpatient in the pediatric intensive care unit. He was admitted two months prior due to hemoptysis, anemia, and respiratory distress. After a diagnosis of idiopathic dilated cardiomyopathy was made, he underwent placement of a left ventricular assist device while awaiting a cardiac transplant. His hospital course was complicated by renal failure, sepsis, pulmonary embolism, heparin-induced thrombocytopenia, pneumothorax, oral candidiasis, and euthyroid sick syndrome. He was intubated, sedated, and mechanically ventilated throughout his hospital stay. Bilateral incomplete eyelid closure (lagophthalmos) was noted by his primary team early during the course of his admission. He was treated intermittently with various strategies such as artificial tears on an as-needed basis, artificial tears every two hours, and erythromycin ointment three times daily. Ophthalmology was eventually consulted due to increasing ocular redness and discharge bilaterally, despite the above therapies. The patient's mother recalled observing intermittent nocturnal lagophthalmos for years prior to his admission. He had never complained of or exhibited any ocular symptoms or signs. External eye examination showed: 4 mm of lagophthalmos (incomplete eyelid closure) OU Apparent loss of Bell's phenomenon OU No proptosis and Portable slit lamp exam showed Eyelids: marked blepharitis and green, sticky mattering OU Conjunctiva/sclera: moderate conjunctival injection OU, especially in the inferior third of the palpebral fissure; mucopurulent ropey discharge OU Cornea: bilateral 6 x 3 mm inferior epithelial defects with central areas of 20% stromal thinning (dellen) and a horizontal linear superior border; some adherent superficial purulent material, but after irrigation, no corneal infiltrates were

noted Anterior chamber: deep; difficult to asses for cell and flare at the bedside; no hypopyon OU. what is the most likely diagnosis?

Case Number 10:

A 57-year-old Caucasian female presented asymptomatically at the age of 46 with bilateral corneal verticillata, early cataracts, and vascular tortuosity. Her primary care provider noted that she had a history of numbness and tingling of her hands and feet that resolved in early adulthood, proteinuria, hypertension, and asymptomatic left ventricular hypertrophy. She subsequently was lost to follow up with ophthalmology until 2017 when she presented with increasing blurry vision with reading and distance over the past 2 years. The patient reports that her last eye exam was 7 years ago and has been wearing the same prescription since. her visual acuity was in Right eye (OD): 20/60-1 Left eye (OS): 20/50-1 and slit lamp examination showed: Lids/lashes: Normal Conjunctiva/sclera: Normal Cornea: Verticillata both eyes (OU) Anterior chamber: Deep and quiet Iris: Normal architecture Lens: 1+ nuclear sclerosis cataract, 1+ cortical cataract.
what is the most likely diagnosis?

Case Number 11:

A 35-year-old female was sent to neuro-ophthalmology for evaluation due to complaint of intermittent blurry vision OU lasting hours. The patient complained of decreased vision upon awakening that is not associated with pain. She stated that it's difficult for her to read. The patient also mentioned that her vision seems to clear up as the day progresses. ocular examination showed: Vision: 20/25 OU at distance and near. Pupils: 5 mm in dark, 2 mm in light, no RAPD. Extraocular Motility: Full motility without pain. IOP: 18 mmHg OU. Visual Field: Full OU. DFE: normal macula, vessels, and periphery OU. SLE: notable for a beaten metal appearance of the corneal endothelium OU.
what is the most likely diagnosis?

Case Number 12:
A 36-year-old female presented to the Emergency Treatment Center (ETC) of the University of Iowa Hospitals and Clinics (UIHC) with one day of right eye pain, photophobia and decreased vision. There was no history of trauma. The ETC physician performed fluorescein staining and made a diagnosis of a corneal abrasion. The patient was treated with topical trimethoprim-polymyxin (Polytrim) four times daily and oral acetaminophen-hydrocodone 10/500 (Lortab) as needed for pain relief. She was instructed to follow-up with her eye care provider the next day if symptoms did not improve or resolve. Slit lamp examination showed: Conjunctiva/Sclera: OD: 1+ conjunctival injection OD without papillary or follicular reaction OS: Normal Cornea: OD: Four small dendritic epithelial defects in the visual axis; no evidence of basement membrane dystrophy; clear and compact stroma without vascularization; no perineuritis; no endothelial inflammation OS: Clear and compact with no evidence of basement membrane dystrophy. **what is the most likely diagnosis?**

Case Number 13:

A 72-year-old female with decreased vision referred for "corneal ulcer" in the right eye (OD). she has Extensive prior ocular issues, including primary open angle glaucoma, neurotrophic keratitis OD, and

multiple bouts of herpes simplex virus (HSV) keratitis in both eyes (OU). Surgically, she had undergone cataract extraction in both eyes (posterior chamber intraocular lens implant in the right eye and aphakic in the left eye) and penetrating keratoplasty in the right eye in 1996. She underwent three penetrating keratoplasties in the left eye (OS) in 1997, 1998 and 2000, and eventually required an evisceration for a blind, painful eye in 2003. ocular examination showed: OD -- epithelial defect (2.6mm vertically by 2.8mm horizontally) centrally in corneal graft. White, arborizing, crystalline infiltrate present in anterior 1/3 of corneal stroma under epithelial defect. Inferior punctual plug in place. There was a rare cell per high power field view in the anterior chamber. Thin, white, mucoid discharge. OS -- Prosthesis, no discharge. **what is the most likely diagnosis?**

Case Number 14

A 68-year-old female was referred by an outside ophthalmologist for evaluation of an anterior stromal corneal dystrophy causing progressive decline in vision with glare over the past 10 years. She complained of having to manually lift her upper eyelids to improve her superior visual field and had not been able to raise her eyebrows for approximately 25 years, a trait also present in both of her sisters. She was unaware if this affected either of her parents or her brother, but states that her father had an unknown problem with his corneas that caused him to have poor vision as well as droopy eyelids. her visual acuity was: Right eye (OD): 20/70 (No improvement with pinhole) Left eye (OS): 20/10 (No improvement with pinhole). slit lamp examination showed: in her right eye ; Salzmann's nodules inferior to visual axis with diffuse anterior stromal haze all the way to the limbus, diffuse punctate epitheliopathy particularly inferiorly in an exposure pattern, reduplicated basement membrane with lattice lines, and her left eye: Salzmann's nodules inferior to visual axis with diffuse anterior stromal haze all the way to the limbus, diffuse punctate epitheliopathy particularly inferiorly in an exposure pattern, reduplicated basement membrane with lattice lines were seen. **what is the most likely diagnosis?**

Case Number 15

A 60-year-old male with a history of simple large cornea presented to the Iowa City Veterans Administration Healthcare System eye clinic reporting visual disturbance while changing head position for several months. He noticed that his vision worsened with his head bent down. He previously had cataract surgery with an iris-sutured IOL due to the large size of his eye, which did not allow for placement of an anterior chamber intraocular lens (AC-IOL) or scleral-fixated lens. ocular examination showed: Visual Acuity (with correction): OD 20/100 (cause unknown) OS 20/20 IOP: 18mmHg OD, 17mmHg OS Slit lamp exam: >13 mm in diameter in cornea and with anterior mosaic dystrophy. Iris-sutured posterior chamber IOLs (PC-IOLs), stable OD, but pseudophacodonesis OS with loose inferior suture evident. **what is the most likely diagnosis?**

Case Number 16:

A 79-year-old woman was referred for the evaluation of a corneal ulcer in the right eye (OD). She began experiencing foreign body sensation in the right eye about 2 months prior to presentation after getting poked in the eye while gardening. Since that time, she had experienced pain, tearing, photophobia, and decreased vision in the eye. The patient was treated with prednisolone acetate 1% drops every 2 hours in the right eye locally, but this was discontinued as corneal thinning was noted. She was a poor historian but apparently carried a diagnosis of "rheumatoid arthritis" for which she chronically took 5 mg of oral (PO) prednisone daily. This was increased to 60mg PO daily by her local ophthalmologist about 2 weeks prior to her referral. She had never seen a rheumatologist. she has a past medical history of rheumatoid arthritis. slit lamp examination showed corneal Crescent-shaped ulceration of peripheral cornea between 11:00 and 3:00 with associated epithelial defect and 80% stromal thinning, conjunctival 1+ injection superiorly, and 3+ cell, trace flare in anterior chamber. **what is the most likely diagnosis?**

Case Number 17:

A three-year-old male who was referred to the cornea clinic after failing a KidSight screening and was found to have bilateral corneal opacities when evaluated by his optometrist. Otherwise the child is in good health without any known medical problems. slit lamp examination showed in both eyes : Diffusely scattered, well-circumscribed areas of stromal haze localized to the posterior 1/3 of the stroma. Most measure about 0.5 mm in size with intervening clear spaces. No associated neovascularization. No epithelial breakdown or erosions. **what is the most likely diagnosis?**

Case Number 18

A 79-year-old female referred from primary ophthalmologist Following cataract surgery with anterior chamber intraocular lens (ACIOL) placement in both eyes (OU) nearly 20 years before. She underwent penetrating keratoplasty (PKP) with intraocular lens exchange in the right eye in 1996. In the years following, the right eye subsequently developed corneal graft failure. The patient had an episode of anterior ischemic optic neuropathy (AION) in this same right eye that further decreased her vision. Functioning thereafter with her left eye, the patient maintained a visual acuity that enabled her to read the newspaper and perform her activities of daily living for several years. However, the patient eventually experienced progressive decline in her vision, OS. The patient began to notice increasing tearing and light sensitivity. Vision deteriorated rapidly in the months prior to her referral to the cornea service at the University of Iowa Hospitals and Clinics. ocular examination showed: Visual Acuity: OD-- Hand motions at 3 feet; OS-- Count fingers at 6 feet Intra-ocular pressure: OD-- 19 mmHg; OS-- 22 mmHg Motility: Orthophoric in primary gaze and full motility, OU. External and anterior segment examination showed the following: Right eye: Full-thickness penetrating keratoplasty

(PKP) graft with severe corneal edema (pachymetry measured 900+ um thick) and a dense paracentral scar. Anterior chamber IOL and correctopia of the pupil Left eye: Edematous cornea (pachymetry measured 826 um thick centrally) and microcysic epithelial changes with folds in Descemet's membrane. There was an IOL in the anterior chamber and 1+ anterior chamber cell. **what is the most likely diagnosis**?

Case Number 19

A 62-year-old woman presents with progressive loss of vision in both eyes Over the last 3 years, there has been gradual, painless loss of vision in both eyes (right greater than left) at both distance and near. She has had 3 updates of her spectacle prescription that have not provided satisfactory vision. There is no diurnal variation in vision. he has a Past Ocular History of Myopia, presbyopia, and astigmatism. Ocular Exam showed: Visual acuity (VA) with correction: Right eye (OD): 20/200, pinhole to 20/50-1 Left eye (OS): 20/25+2, pinhole to 20/20 Manifest Refraction: OD -2.00 + 5.00 x 20 VA 20/60 OS -0.75 + 7.50 x 165 VA 20/25 slit lamp examination of Cornea showed: OD: Greyish nodules between the corneal epithelium and Bowman’s layer in the paracentral superonasal region extending centrally into the visual axis; iron line at inferior border of the lesion OS: Greyish nodules between the corneal epithelium and Bowman’s layer in the paracentral superior and superotemporal regions extending centrally into the visual axis; iron line at inferior border of the lesion**. what is most likely the diagnosis?**

Case Number 20

A 54-year-old white male with a known history of atrial fibrillation and hypertension. The patient had been on Amiodarone therapy for several years. Two months prior to this routine follow up, the patient's primary care doctor made a change in the patient's cardiac medications which included the removal of amiodarone from his medication regimen. The patient has no vision complaints. slit lamp examination showed notable for corneal deposits in the cornea at the level of the basal epithelium, inferiorly OU. Deposits form a faint golden-brown whorl pattern evident in both corneas. **what is the most likely diagnosis?**
